# Pathophysiology, Evaluation, and Management of Metabolic Alkalosis

**DOI:** 10.7759/cureus.12841

**Published:** 2021-01-21

**Authors:** Mohammad Tinawi

**Affiliations:** 1 Nephrology, Nephrology Specialists, P.C, Munster, USA; 2 Medicine, Indiana University School of Medicine Northwest, Gary, USA

**Keywords:** metabolic alkalosis, acid-base disorders, acid-base physiology, alkalemia

## Abstract

Metabolic alkalosis is an increase in blood pH to >7.45 due to a primary increase in serum bicarbonate (HCO_3_^−^). Metabolic alkalosis results from alkali accumulation or acid loss, and it is associated with a secondary increase in carbon dioxide arterial pressure (P_a_CO_2_). Metabolic alkalosis is a common acid-base disorder, especially in critically ill patients. The pathogenesis of chronic metabolic alkalosis includes two derangements, generation of metabolic alkalosis via gain of alkali or loss of acid and maintenance of metabolic alkalosis by increased tubular HCO_3_^−^ reabsorption (failure of the kidneys to excrete excess alkali). Metabolic alkalosis is the most common acid-base disorder in hospitalized patients, particularly in the surgical critical care unit. Mortality increases as pH increases.

## Introduction and background

The focus of this review article is the pathophysiology of metabolic alkalosis, as well as its causes, diagnosis, and management. The PubMed database was searched for relevant basic science and clinical articles in addition to the leading journals in nephrology, endocrinology, critical care, and internal medicine. The articles reviewed included clinical trials, comprehensive reviews, and case studies deemed of clinical significance. Chapters from the major textbooks were reviewed as well.

Intracellular pH is 7.0-7.30 while normal arterial blood pH is 7.35-7.45 [1-2]. Arterial blood pH is kept in a narrow range due to renal and respiratory regulations and multiple intracellular and extracellular buffers. Arterial blood gases (ABGs) are required to ascertain the diagnosis of acid-base disorders. For example, high serum HCO3^−^ can result from metabolic alkalosis or metabolic compensation for respiratory acidosis. A high blood pH >7.45, i.e., low arterial blood hydrogen (H+) defines alkalemia, if serum HCO3^− ^is high, the alkalemia is due to metabolic alkalosis, while if carbon dioxide arterial pressure (P_a_CO_2_) is low, the alkalemia is due to respiratory alkalosis. In metabolic alkalosis, arterial HCO3^−^ is >28 mmol/l and venous total CO_2_ is >30 mmol/l [3]. A gain of alkali or loss of acid leads to metabolic alkalosis. If serum HCO3^−^ is high and P_a_CO_2 _is low, the alkalemia is due to a mixed acid-base disorder, namely, metabolic alkalosis and respiratory alkalosis [4]. A simple acid-base disorder is due to a change in either P_a_CO_2_ or serum HCO3^− ^with appropriate metabolic or respiratory compensation, respectively. A mixed acid-base disorder is the presence of two or three acid-base disorders simultaneously [5]. Metabolic alkalosis is usually accompanied by hypokalemia and hypochloremia. This reduction in chloride (Cl^-^) is not accompanied by hyponatremia [6]. Serum anion gap (AG) is usually slightly elevated in metabolic alkalosis due to an increase in the net negative charges of plasma proteins [7]. To summarize, in simple acid-base disorders, serum HCO3^−^ and P_a_CO_2_ move in the same direction (both are up in metabolic alkalosis and respiratory acidosis and both are down in metabolic acidosis and respiratory alkalosis). In mixed acid-base disorder, serum HCO3^− ^and P_a_CO_2_ move in the opposite direction.

The next step after diagnosing metabolic alkalosis is the determination of respiratory compensation. For every 1 mmol/l rise in HCO3^−^ above 24 mmol/l, there is a 0.6 mmHg rise in P_a_CO_2_ as per the following equation:

P_a_CO_2_ (mmHg) = 40 + 0.6 × (HCO3^−^ - 24 mmol/l)

For example, if HCO3^−^ is 40 mmol/l, the rise in HCO3^−^ is 40-24 = 16 mmol/l, the rise in P_a_CO_2_ is 0.6 x 16 = 9.6 mmHg, and the expected P_a_CO_2_ is 40 + 9.6 or approximately 50 mmHg. A quick way to determine P_a_CO_2_ is by adding 15 to HCO3^−^ [8]. Metabolic alkalosis is accompanied by alveolar hypoventilation, which takes minutes to hours to occur [9]. Respiratory compensation usually does not result in complete pH normalization [9]. In metabolic alkalosis, P_a_CO_2_ is rarely over 55 mmHg. A pH that is close to the normal range may indicate a mixed acid-base disorder, namely, metabolic alkalosis and respiratory acidosis [10]. An example of such disorder is a patient with chronic respiratory acidosis due to chronic obstructive pulmonary disease (COPD) who develops a concomitant metabolic alkalosis due to diuresis. It is helpful to remind the reader of the concept of base excess, which is routinely reported on ABGs. An ABG sample under standard (normal) conditions has a pH of 7.40, P_a_CO_2_ of 40 mmHg, a temperature of 37°C, and a base excess of 0 mmol/l. Base excess is defined as the amount of strong acid in mmol/l that needs to be added to one liter of fully oxygenated blood in vitro to return an ABG sample to the above-defined standard conditions [11]. Base excess is negative in metabolic acidosis and positive in metabolic alkalosis. The normal range of base excess is -2 to +2 mmol/l. For example, an ABG sample in a patient with severe metabolic alkalosis showed pH 7.55, P_a_CO_2_ 49 mmHg, HCO3^−^ 38 mmol/l, and a base excess of 14 mmol/l.

## Review

Incidence

Metabolic alkalosis was the most common acid-base disorder in patients in the intensive care unit (ICU) in a large Norwegian study [12]. The study analyzed 138,523 ABGs. On admission to ICU, acidosis (metabolic and respiratory) is more common. HCO3^−^ increases over time. Alkalosis was defined as base excess > 0 on ABGs and was found in 118,014 samples (85%). A stricter definition of alkalosis as base excess > 2 mmol/L would have led to a lower reported incidence. Alkalosis was found as a simple or mixed acid-base disorder, including post-hypercapnic alkalosis in patients with COPD on mechanical ventilation. A prospective study by Okusawa et al. enrolled 293 general surgical patients [13]. Six ABGs were taken from each patient starting on postoperative day 0 and ending on postoperative day 7. The vast majority of patients (87.5%) had a normal acid-base balance preoperatively. Postoperatively, 50.5% of patients developed metabolic alkalosis. Other acid-base abnormalities were uncommon. Metabolic alkalosis persisted in 31 patients and carried a high mortality rate of 32%. The administration of fresh frozen plasma (FFP) was a major cause of metabolic alkalosis postoperatively. Hodgkin et al. analyzed 13,430 ABGs obtained from hospitalized patients [14]. Metabolic alkalosis was by far the most common acid-base disorder (51%), followed by respiratory alkalosis (29%), then respiratory acidosis (27%), and, finally, metabolic acidosis (12%). The reported incidence adds to more than 100% due to the presence of mixed acid-base disorders in some patients. For example, metabolic alkalosis was simple in 70% of cases and mixed with respiratory or metabolic acidosis in the remaining 30%. An earlier study by Wilson et al. in 1415 critically ill surgical patients showed that 177 (12%) developed severe metabolic alkalosis defined as arterial pH >7.54 [15]. More severe metabolic alkalosis was associated with higher mortality. Mortality was 41% in patients with pH 7.55-7.56, 47% in patients with pH 7.57-7.59, 65% in patients with pH 7.60-7.64, and 80% in patients with pH 7.65-7.70. A prospective study by Anderson et al. in a group of 409 medical and surgical patients showed that mortality was 48.5% in patients with pH >7.60 [16].

Pathophysiology of metabolic alkalosis

**Figure 1:**
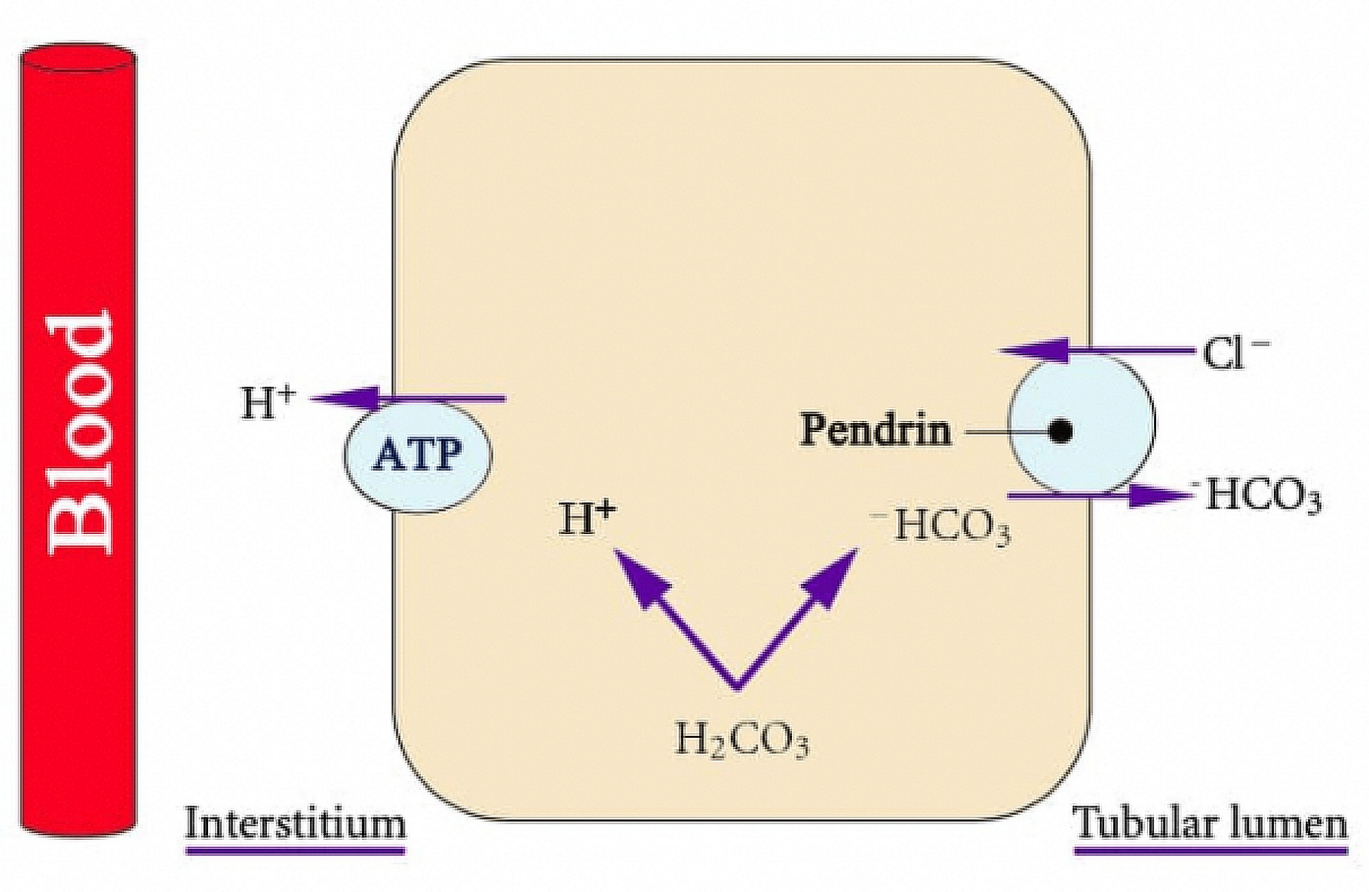
Pendrin is an apical chloride-bicarbonate exchanger in beta-intercalated cells ATP: adenosine triphosphate Courtesy of Bruno and Valenti. J Biomed Biotechnol. 2012 [19]. This is an open-access article distributed under the Creative Commons Attribution License.

The kidneys play a major role in acid-base regulation. The three components of renal net acid excretion are ammonium (NH4+), titratable acid, and urinary HCO3^−^ ( U_HCO3^−^_) [1-2]. All of the HCO3^−^ filtered through the glomeruli is reabsorbed under normal physiological conditions [17]. The kidneys generate new HCO3^−^ to replace the HCO3^−^ used to buffer acid in the body. About 80% of filtered HCO3^−^ is absorbed by the proximal tubule (PT) while the thick ascending limb (TAL) of the loop of Henle reabsorbs 15%. The cortical collecting duct (CCD) and the inner medullary collecting duct (IMCD) reabsorb the remaining 5% [1]. The two cell types of the CCD are the intercalated cells, which regulate acid-base balance, and the principal cells, which, under the effect of aldosterone, secrete potassium (K^+^), and reabsorb sodium (Na^+^). There are two subtypes of intercalated cells (they are the functional mirror images of each other), alpha-intercalated cells, which secrete H^+^, and beta-intercalated cells, which secrete HCO3^−^ in exchange for Cl^-^ and, therefore, play a role in the correction of metabolic alkalosis. The medullary collecting duct does not contain beta-intercalated cells [1-2]. In the alpha-intercalated cells, the generated HCO3^−^ exits the cell via a basolateral Cl^-^-HCO3^−^ exchanger. The beta-intercalated cells generate HCO3^−^, which exits the cell and enters the tubular lumen via an apical Cl^-^-HCO3^−^ exchanger (SLC26A4 protein {pendrin}) [18-19] (Figure 1). HCO3^−^ secretion in the collecting duct (CD) requires luminal Cl^-^ and is inhibited by Cl^- ^depletion. As we shall see, Cl^-^ depletion is critical in the generation of metabolic alkalosis [3]. Cl^-^depletion increases distal Na^+^ delivery and reabsorption, which stimulates K^+^ and H^+^ secretion. This explains the importance of isotonic saline solutions in the correction of metabolic alkalosis. The density of beta-intercalated cells and pendrin are both reduced in the case of hypokalemia [20]. Therefore, hypokalemia limits HCO3^−^ excretion, which explains the importance of the correction of hypokalemia in the management of metabolic alkalosis.

Two steps are required for metabolic alkalosis to persist. The first step is the generation of metabolic alkalosis and the second step is the maintenance of metabolic alkalosis [21]. In other words, when faced with metabolic alkalosis, one has to answer two questions. First, what is the source of the excess HCO3^− ^and second, why is the excess HCO3^− ^not excreted by the kidneys?

Generation of Metabolic Alkalosis

The generation of metabolic alkalosis is due to excess HCO3^−^ accumulation originating from endogenous or exogenous sources (Table 1).

**Table 1:** Exogenous and endogenous mechanisms resulting in the generation of metabolic alkalosis

Infusion or ingestion of HCO_3_^−^ (e.g. NaHCO_3_) or HCO_3_^−^ precursors (citrate, lactate, or acetate)
Hydrochloric acid (HCl) loss as in vomiting, Cl^-^-rich diarrhea, or nasogastric (NG) suction
Hypokalemia leads to extracellular K^+ ^shift balanced by concomitant intracellular H^+^ shift
Excess HCO_3_^−^ generation by the distal tubule (due to increased H^+ ^excretion) due to increased distal delivery and subsequent absorption of Na^+^ as in primary hyperaldosteronism, use of thiazide or loop diuretics, Gitelman and Bartter syndromes, and infusion of Na penicillin (penicillin is a poorly absorbed anion)

An example of exogenous ingestible Na^+^ and K^+^ alkali salts include baking soda (60 mmol/teaspoon), NaHCO3 tablets (7.8 mmol/650 mg tablet), KHCO3 tablets, K citrate tablets, Polycitra-K® and Cytra-2®. Na citrate is added as an anticoagulant to whole blood or fresh frozen plasma (FFP) and can be an important source of alkali in patients needing massive amounts of blood products as in those treated with large dose plasma exchange [9]. HCO3^−^ is generated upon complete oxidation of citrate, acetate, and lactate, all of which are organic anions. Loss of HCl due to vomiting or NG suctioning results in HCO3^− ^generation [8]. It is useful to recall that loss of acid (H^+^) is equivalent to the gain of alkali (HCO3^−^) and vice versa. Infusion of large amounts of Na penicillin or other Na+ salts of nonreabsorbable anions, such as sulfate or phosphate, will generate HCO3^−^ if Na+ reabsorption via the distal tubule is enhanced by volume depletion or mineralocorticoids (aldosterone) [9,22]. Na^+^ is reabsorbed while HCO3^−^ is generated and H^+^ is excreted as titratable acid or NH4^+^.

Maintenance of Metabolic Alkalosis

Under normal circumstances, the kidneys excrete excess HCO3^−^ and restore the acid-base balance. Normal kidneys have an enormous ability to excrete large amounts of HCO3^−^ when ingested chronically [23]. Some advocate the use of NaHCO3 to improve athletic performance. When NaHCO3 was given progressively and chronically in amounts up to 400 mg/kg, it was well-tolerated [23-24]. The failure of the kidneys to excrete excess HCO3^−^ is due to the presence of a mechanism that leads to the maintenance of metabolic alkalosis (Table 2).

**Table 2:** Mechanisms of maintenance of metabolic alkalosis

Decreased HCO_3_^−^ filtration due to a decline in glomerular filtration rate (GFR)
Increased HCO_3_^−^ reabsorption in the proximal tubule due to volume (and Cl^-^) depletion
Increased H^+^ secretion and NH_4_^+^ excretion due to hypokalemia or increased aldosterone

Some clinical events activate more than one mechanism for the maintenance of metabolic alkalosis. For example, volume (and Cl^-^) depletion decreases HCO3^−^ filtration due to a decrease in GFR, increases proximal tubular reabsorption of HCO3^−^, and enhances H^+^ secretion and NH4^+ ^excretion due to increased aldosterone and subsequent hypokalemia [8].

Etiology of Metabolic Alkalosis

Metabolic alkalosis is divided into two major categories base on extracellular fluid (ECF) volume status, metabolic alkalosis with ECF volume contraction, and metabolic alkalosis with ECF volume expansion [8-9]. Urine chloride (Ucl^-^) is helpful in differentiating the two categories. Ucl^-^ is <20 mmol/l in ECF volume contraction and Ucl^-^ ≥20 mmol/l in ECF volume expansion (Table 3). Contraction alkalosis occurs whenever there is ECF volume depletion (contraction) associated with a fixed amount of HCO3^−^.

**Table 3:** Causes of metabolic alkalosis

ECF volume contraction	ECF volume expansion
Vomiting, nasogastric (NG) suction	Primary aldosteronism
Congenital chloridorrhea (Cl^-^-rich)	Renal artery stenosis
Villous adenoma	Renin-secreting tumors
High volume ileostomy output	Glucocorticoid remediable aldosteronism
Post-hypercapnic state	Cushing’s syndrome or disease
Thiazide or loop diuretics (U_cl_^-^ is variable)	Exogenous mineralocorticoids
Cystic fibrosis associated with severe perspiration	Congenital adrenal hyperplasia due to 11-beta or 17-alpha hydroxylase deficiency
Bartter syndrome	Licorice (reduced activity of 11-beta hydroxysteroid dehydrogenase )
Gitelman syndrome	Liddle syndrome

Other important causes of metabolic alkalosis in which volume status is variable include, hypokalemia, hypomagnesemia, milk-alkali (calcium-alkali) syndrome (where many patients have reduced GFR limiting HCO3^−^excretion), alkali load especially with reduced GFR, nonreabsorbable anions, such as penicillin and carbenicillin, and refeeding post fasting or starvation [25].

Typical causes of metabolic alkalosis associated with ECF volume contraction are vomiting and NG suction. The gastric H^+^-K^+^ ATPase secretes HCl into the stomach lumen. When HCl reaches the small bowel, it is neutralized by an equal amount of HCO3^−^ secreted into the lumen of the small bowel. The removal of gastric HCl due to vomiting or nasogastric (NG) suction results in metabolic alkalosis because HCO3^−^ is added to the ECF (rather than secreted into the small bowel lumen). HCO3^−^ is subsequently excreted by the kidneys as NaHCO3, resulting in volume depletion [9]. Metabolic alkalosis is maintained in this case due to volume depletion, secondary aldosteronism, loss of K^+^ (due to secondary aldosteronism and increased distal delivery of NaHCO3). Moreover, hypokalemia shifts K+ extracellularly and subsequently H^+^ (a positive cation) intracellularly [26]. Intracellular acidosis stimulates further HCO3^−^ generation. U_cl^-^_,_^ ^_in this case, is <20 mmol/l, while U_Na^+^ _can be elevated due to the excretion of Na^+^ with HCO3^−^ by the kidneys. Diarrhea usually results in non-anion gap metabolic acidosis. Villous adenoma results in metabolic alkalosis due to loss of K^+^ in stool and volume depletion. Likewise, congenital chloridorrhea also results in metabolic alkalosis. The latter is a rare autosomal recessive disorder due to mutation in the gene SLC26A3 resulting in loss of function of the ileal HCO3^−^-Cl^- ^exchanger and subsequent HCO3^−^ retention [27]. Thiazide and loop diuretics can result in metabolic alkalosis with ECF volume contraction and hypokalemia due to enhanced distal delivery of water and Na^+^ and secondary hyperaldosteronism [28]. Ucl^-^_ _is variable, it is elevated (≥20 mmol/l) when diuretics are working and low (<20 mmol/l) when their effect wears off. Bartter syndrome is due to a loss of function mutation in the TAL resulting in effects similar to the use of a loop diuretic. Gitelman syndrome is due to loss of function mutation of the Na^+^-Cl^-^ cotransporter in the distal collecting duct (DCT) mimicking the effects of a thiazide diuretic [29]. Liddle syndrome is a rare genetic cause of severe hypertension due to gain of function mutation of the epithelial sodium channel (ENaC) resulting in hypokalemia and hyporeninemic hypoaldosteronism [8]. Ucl- is elevated (≥20 mmol/l) in Bartter and Gitelman syndromes. In patients with acute respiratory acidosis, HCO3^−^ goes up by 1 mmol/l for each 10 mmHg increase in P_a_CO_2_, while in chronic respiratory acidosis, HCO3^−^ goes up by 4 mmol/l for each 10 mmHg increase in P_a_CO_2_. For example, a patient with chronic respiratory acidosis and a P_a_CO_2_ of 70 mmHg, is expected to have serum HCO3^−^ of 36 mmol/l (24 +{4x3}) due to metabolic compensation. In the post-hypercapnic state, the respiratory acidosis has improved (as in COPD patients who are placed on mechanical ventilation) but the elevation of HCO3^−^ persists, resulting in metabolic alkalosis [30]. Excessive perspiration can cause metabolic alkalosis in patients with cystic fibrosis (CF) [31]. Unexplained metabolic alkalosis with volume depletion and hyponatremia should raise the possibility of cystic fibrosis; this presentation is more common during a heatwave. Aminoglycoside use in patients with CF can result in metabolic alkalosis due to a Bartter-like syndrome. Aminoglycosides activate the calcium-sensing receptor (CaSR), resulting in inhibition of the Na^+^-K^+^-2Cl^-^ transporter in the TAL with subsequent increase in urinary Na^+^, K^+^, Ca^+2^, and Mg^+2^ [32-33]. The intact PTH level is low despite hypocalcemia due to the activation of CaSR. A typical cause of metabolic alkalosis associated with ECF volume expansion is primary aldosteronism commonly caused by unilateral aldosterone-secreting adenoma or adrenal hyperplasia. Adrenal carcinoma is rare. Primary aldosteronism manifestations are hypertension, hypokalemia, metabolic alkalosis, and ECF volume expansion [9]. Distal Na^+^ reabsorption is enhanced by aldosterone, resulting in ECF volume expansion. This, in turn, increases K^+ ^excretion resulting in hypokalemia. HCO3^−^ generation and retention are increased with hypokalemia as explained above [26]. Metabolic alkalosis is maintained due to autonomous aldosterone secretion. Ucl- in this case is >20 mmol/l due to ECF volume expansion [29].

Diagnosis of Metabolic Alkalosis

Metabolic alkalosis is an elevation in blood pH to >7.45. ABGs are required to ascertain the diagnosis of acid-base disorders because high serum HCO3^−^ can result from metabolic alkalosis or metabolic compensation for respiratory acidosis. History can identify potential causes of metabolic alkalosis such as vomiting, diuretic use, licorice intake, cystic fibrosis, exogenous sources of HCO3^−^, or primary aldosteronism. Physical examination helps in evaluating ECF volume status. Most clinicians make the diagnosis of metabolic alkalosis in appropriate clinical settings (such as vomiting, NG suction, primary aldosteronism) based on history, physical exam, and basic chemistry profile without doing ABGs. A basic chemistry profile is needed for the diagnosis of metabolic alkalosis. Measurement of other electrolytes (other than serum HCO3^−^), including Na^+^, K^+^, Cl^-^, and Mg^+2^, is critical. Urea and creatinine help in evaluating renal function. Urine electrolytes are obtained. Ucl^-^ is elevated (≥20 mmol/l) in case of ECF volume expansion and low (<20 mmol/l) in case of ECF volume contraction. Obtaining Ucl^-^ concentration from a random urine sample is adequate and 24-hour urine collection is usually not necessary. Patients suspected of having primary aldosteronism require further testing [34]. Surreptitious diuretic use is an important cause of metabolic alkalosis. Varying and parallel levels of UNa^+^ and Ucl^-^ (both are high on some occasions and low on other occasions) raise the possibility of diuretic abuse. Urine diuretic screening is confirmatory. Bartter and Gitelman syndromes require genetic testing to ascertain the diagnosis. The diagnosis of congenital adrenal hyperplasia due to 11-beta or 17-alpha hydroxylase deficiency requires a specialist consultation [35]. Pendred syndrome is an autosomal recessive disorder characterized by deafness and thyroid goiter. It is due to mutations in gene SLC26A4. SLC26A4 encodes for pendrin, which is expressed by the thyroid gland, the inner ears, and the apical membrane of beta-intercalated cells in the cortical collecting duct in the kidneys. Pendred syndrome patients do not have renal abnormalities under basal conditions. They are at risk of developing life-threatening metabolic alkalosis in case of volume depletion or treatment with thiazide diuretics [36]. If a thiazide diuretic is added to acetazolamide, a profound salt-wasting ensues because acetazolamide downregulates pendrin [37].

Manifestations of Metabolic Alkalosis

Most patients with mild to moderate metabolic alkalosis are asymptomatic. Serum HCO3^−^ of up to 40 mmol/l is tolerated. The manifestations are usually due to concomitant hypokalemia, hypophosphatemia, hypoventilation, volume depletion, and hypocalcemia. Metabolic alkalosis decreases ionized calcium levels [33]. Severe metabolic alkalosis (serum HCO3^− ^>45 mmol/l) can lead to tetany, seizures, cardiac arrhythmias, and delirium [3]. Alkalemia also inhibits respiratory drive and shifts the oxygen-hemoglobin dissociation curve to the left.

Management of Metabolic Alkalosis

In patients with metabolic alkalosis associated with volume contraction (Cl^-^-sensitive metabolic alkalosis), it is critical to give isotonic saline (0.9 NaCl) and to replete potassium with potassium chloride orally (PO), intravenously (IV), or both [38]. This strategy will lead to NaHCO3 diuresis and the restoration of the acid-base balance. Mg^+2^ should be replaced in patients with hypomagnesemia because hypomagnesemia can result in recalcitrant hypokalemia [38]. Diuretics should be discontinued if feasible; otherwise, the diuretic doses should be reduced. In some patients, such as those with heart failure or liver cirrhosis, discontinuation of diuretics is not an option. In those cases, the addition of a K^+^ sparing diuretic, such as spironolactone, eplerenone, amiloride, or triamterene, may be helpful because it mitigates hypokalemia and hypomagnesemia. Acetazolamide is bicarbonaturic, however, it is very kaliuretic necessitating aggressive K+monitoring and replacement [28]. It should only be utilized by experienced clinicians. It can be given orally (PO) or intravenously (IV) at a dose of 250-500 mg, two to three times daily. The Diabolo study was a double-blind, randomized trial conducted in 15 intensive care units (ICUs) in France [39]. It involved 382 patients with COPD on mechanical ventilation with pure or mixed metabolic alkalosis. Patients in the active arm were given a large dose of acetazolamide (500-1000 mg) twice daily IV. Acetazolamide did not change the primary outcome, which was the reduction in the duration of invasive mechanical ventilation via endotracheal intubation or tracheotomy. There was a statistically significant small reduction in HCO3^−^(0.8 mmol/l) in the acetazolamide group.

Patients with metabolic alkalosis with ECF volume expansion (Cl^-^-resistant metabolic alkalosis) should have their K^+ ^replaced as well. The underlying etiology, such as adrenal adenoma, should be the main focus of treatment. Patients with bilateral adrenal hyperplasia are treated with aldosterone blockers such as spironolactone or eplerenone. A low Na^+^ diet is helpful in patients with hyperaldosteronism because it reduces distal Na+ delivery. Peritoneal dialysis or hemodialysis (with a low HCO3^−^ in the dialysate {30-32 mmol/l}) are helpful in correcting metabolic alkalosis in patients with advanced chronic kidney disease (CKD) or who are already on dialysis [40]. Continuous renal replacement therapy (CRRT) is particularly helpful in the management of severe metabolic alkalosis due to the ability to modify electrolytes in replacement solutions and dialysate [3]. Prolonged exposure to inappropriately high dialysate HCO3^−^ is associated with increased mortality due to post-dialysis metabolic alkalosis [41]. Currently, HCO3^−^ in the dialysate is kept around 35 mmol/l. Infusion of dilute HCl (0.1 normal HCl, 0.1N HCl = 100 mmol/l H^+^) or ammonium chloride (NH4Cl) is rarely done [42]. HCl should be placed in a glass container and infused through a central venous catheter; it can cause severe hemolysis and venous thrombosis and should be discontinued once pH is around 7.50. IV tubing has to be changed every 12 hours. NH4Cl is metabolized into urea and HCl and is associated with central nervous system toxicity and gastrointestinal adverse reactions. NH4Cl can be also given orally. Arginine-HCl is no longer used because it may cause life-threatening hyperkalemia due to the extracellular shift of K^+^.

The etiology of metabolic alkalosis should be addressed. Patients with vomiting should be treated symptomatically and the cause of vomiting should be investigated. The use of proton pump inhibitors (PPIs) or H2 blockers may be helpful in patients with ongoing gastric fluid losses [43]. Exogenous sources of alkali should be identified. Patients should be instructed to avoid licorice or licorice-containing tobacco products. Bartter syndrome is treated with K^+^ repletion, K^+^-sparing diuretics, such as spironolactone and amiloride, and nonsteroidal anti-inflammatory drugs (NSAIDs) due to their prostaglandin blocking effect [29]. Gitelman syndrome is treated in a similar manner in addition to Mg^+2^ repletion. Blanchard et al. evaluated treatment with indomethacin, eplerenone, or amiloride in 30 patients with Gitelman syndrome [44]. Indomethacin was the most efficacious with a 0.38 mmol/l increase in plasma K^+^, followed by amiloride (0.19 mmol/l increase), and then eplerenone (0.15 mmol/l). Forty percent of patients receiving indomethacin discontinued treatment due to gastrointestinal adverse reactions.

## Conclusions

Metabolic alkalosis is the most common acid-base disorder in hospitalized patients, and it is associated with increased mortality. It is generated by a gain of alkali or loss of acid and is maintained by the failure of the kidneys to excrete excess alkali. Metabolic alkalosis is either associated with volume depletion or volume expansion. Volume depletion metabolic alkalosis is Cl^-^-sensitive and is treated with isotonic saline solutions and K^+^ replacement while volume expansion metabolic alkalosis is Cl^-^-insensitive and is treated with K^+^ repletion and by addressing the underlying cause. Recently, the molecular mechanisms of several genetic disorders associated with metabolic alkalosis have been elucidated such as the Bartter, Gitelman, Liddle, and Pendred syndromes.
